# Gap Junctions in the Nervous System: Probing Functional Connections Using New Imaging Approaches

**DOI:** 10.3389/fncel.2018.00320

**Published:** 2018-09-19

**Authors:** Ao Dong, Simin Liu, Yulong Li

**Affiliations:** ^1^State Key Laboratory of Membrane Biology, Peking University School of Life Sciences, Beijing, China; ^2^PKU-IDG/McGovern Institute for Brain Research, Beijing, China; ^3^Peking-Tsinghua Center for Life Sciences, Beijing, China

**Keywords:** gap junction, electrical synapse, fluorescence imaging, genetically encoded methods, nervous system

## Abstract

Gap junctions are channels that physically connect adjacent cells, mediating the rapid exchange of small molecules, and playing an essential role in a wide range of physiological processes in nearly every system in the body, including the nervous system. Thus, altered function of gap junctions has been linked with a plethora of diseases and pathological conditions. Being able to measure and characterize the distribution, function, and regulation of gap junctions in intact tissue is therefore essential for understanding the physiological and pathophysiological roles that gap junctions play. In recent decades, several robust *in vitro* and *in vivo* methods have been developed for detecting and characterizing gap junctions. Here, we review the currently available methods with respect to invasiveness, signal-to-noise ratio, temporal resolution and others, highlighting the recently developed chemical tracers and hybrid imaging systems that use novel chemical compounds and/or genetically encoded enzymes, transporters, channels, and fluorescent proteins in order to map gap junctions. Finally, we discuss possible avenues for further improving existing techniques in order to achieve highly sensitive, cell type-specific, non-invasive measures of *in vivo* gap junction function with high throughput and high spatiotemporal resolution.

## Introduction

Multicellular organisms rely on cell-cell communication to coordinate a wide range of physiological processes and maintain homeostasis. Most organisms have evolved a rich diversity of mechanisms to achieve this communication, including long-distance signaling through the release, and binding of hormones (Ansar Ahmed et al., 1985; Giustina and Veldhuis, 1998; Meier and Gressner, 2004), spatially confined synaptic transmission between two neurons (Krnjevic, 1974; Pereda, 2014), and gap junctional coupling between neighboring cells (Kumar and Gilula, 1996; Sohl et al., 2005; Mese et al., 2007). In the central nervous system, billions of neurons are intermingled and communicate with each other through a specialized structure called the synapse, forming a complex signaling network. Although synapses are predominantly chemical in nature, with neurotransmitters released from the presynaptic terminal and sensed by the postsynaptic neuron via surface receptors, gap junction–based electrical synapses are also widely distributed, and play an essential role in regulating both the development and function of the nervous system (Pereda, 2014).

Gap junctions, composed of connexins in vertebrates and innexins in invertebrates, are intercellular channel complexes between connected cells (Kumar and Gilula, 1996; Phelan et al., 1998). Pannexins are vertebrate homologs to the innexins (Baranova et al., 2004), form hemi-channels connecting cytosol and extracellular space (D’hondt et al., 2009), and could mediate gap junctional connection in cells when overexpressed (Bruzzone et al., 2003; Vanden Abeele et al., 2006; Lai et al., 2007; Ishikawa et al., 2011), although their *in vivo* role in forming functional gap junction is unclear (Sosinsky et al., 2011). Ions and other small molecules with a molecular mass up to approximately 1 to 2 kDa can freely diffuse through gap junctions (Loewenstein, 1981; Kumar and Gilula, 1996; Neijssen et al., 2005). Thus, signals such as action potentials can propagate directly between gap junction–coupled neurons, resulting in virtually no delay in signal transmission (Furshpan and Potter, 1957;Bennett and Zukin, 2004); in contrast, signal transmission via a chemical synapse has a delay on the order of milliseconds (Katz and Miledi, 1965; Sabatini and Regehr, 1996). Gap junctions therefore allow organisms to respond extremely rapidly under certain conditions, for example in the escape reflex in crayfish (Antonsen and Edwards, 2003) and the retina’s response to visual stimuli in vertebrates (Bloomfield and Volgyi, 2009). Gap junctions are also expressed in glial cell types, including astrocytes (Wallraff et al., 2004), microglia (Garg et al., 2005), oligodendrocytes and Schwann cells (Nualart-Marti et al., 2013), and insect blood-brain barrier glial cells (Speder and Brand, 2014), which is essential for the buffering of ions and transmitters, inflammatory response, myelination and neural stem cell proliferation. Gap junctions also connect glia and neurons (Dobrenis et al., 2005; Meng et al., 2016). Given their highly varied and important roles, it is therefore not surprising that malfunctions in gap junctions can disrupt communication among neurons and glia, thus giving rise to a variety of diseases and neurological disorders, including hereditary deafness (Martinez et al., 2009), uncorrelated motor neuron firing (Personius et al., 2007), and Charcot-Marie-Tooth disease (Kleopa, 2011).

**FIGURE 1:**
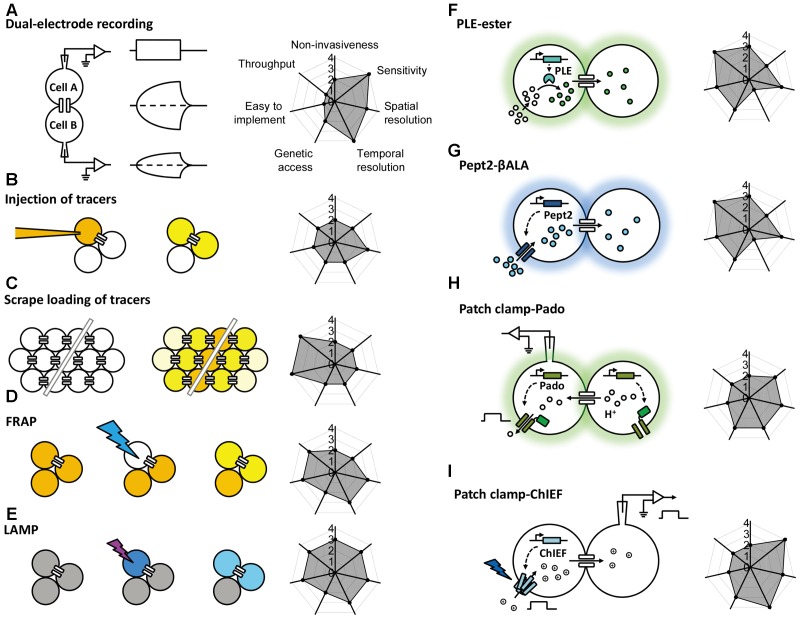
Schematic overview of the currently available methods for studying gap junctions. The principle behind each method is shown schematically on the left with a radar graph on the right that summarizes its corresponding performance index (e.g., sensitivity, throughput, resolution) in arbitrary units ranging from 0 to 4. Further details are provided in the text. FRAP, fluorescence recovery after photobleaching; LAMP, local activation of a molecular fluorescent probe; PLE, porcine liver esterase; Pept2, peptide transporter 2; βALA, AMCA-labeled dipeptides β-Ala-Lys; ChIEF, an engineered version of a channelrhodopsin.

Extensive studies of gap junctions in the nervous system have been carried out by various research groups over the past few decades; the expression of gap junction forming subunits were detected by northern blot (Paul et al., 1991; White et al., 1992), RT-PCR (Wrenzycki et al., 1996; Xia et al., 1998), western blot (Stauffer, 1995; Giepmans and Moolenaar, 1998), and immunohistochemistry (Beyer et al., 1989; Dermietzel et al., 1989). In this review, we focus on functional methods that can detect gap junctional coupling, first briefly summarizing current approaches relying on electrophysiological recording, tracer-based assays, and hybrid methods using genetic tools (**Figure 1**), mainly focusing on recently developed imaging methods. We summarize the performance and properties of these methods, including their invasiveness, throughput, feasibility, sensitivity, spatial resolution, and temporal resolution (**Table 1**). As new *in vivo* methods are being developed, new features of gap junction regulation will likely be revealed, yielding important new insights into the role that gap junctions play in both health and disease.

**Table 1:** Overview of the methods used to probe gap junctional communication.

	Property/characteristic						
Method	Sensitivity	Throughput	Ease of implementation	Genetic access	Temporal resolution	Spatial resolution	Invasiveness
Dual-electrode recording	+ ++	Two cells each time	Technically demanding	No	Milliseconds	Cellular level	Invasive
Injection of tracers	+	Limited number of cells	Technically demanding	No	5–20 min	Cellular level	Invasive
Scrape loading of tracers	+	Dozens of cells	Relatively easy	No	2 min	Cellular level	Invasive
FRAP	+	Dozens of cells	High-power laser	No	∼50 s	Cellular level	Photo damage
LAMP	+ +	Dozens of cells	Relatively easy	No	∼200 s	Cellular level	Non-invasive
PLE-ester	+	Dozens of cells	Relatively easy	Yes	Hours	Cellular level	Non-invasive
Pept2-βALA	+	Dozens of cells	Relatively easy	Yes	Hours	Cellular level	Non-invasive
Patch clamp-Pado	+ +	Limited number of cells	Technically demanding	Yes	Sub-second	Cellular level	Invasive
Patch clamp-ChIEF	+ ++	Limited number of cells	Technically demanding	Yes	Milliseconds	Cellular level	Invasive


*FRAP, fluorescence recovery after photobleaching; LAMP, local activation of a molecular fluorescent probe; PLE, porcine liver esterase; Pept2, peptide transporter 2; βALA, AMCA-labeled dipeptides β-Ala-Lys; ChIEF, an engineered version of a channelrhodopsin.*

## Electrophysiological Recording

Gap junctional coupling can be measured using dual-electrode whole-cell current-clamp recordings (Furshpan and Potter, 1959). This method requires two microelectrodes; one electrode is used to inject current into one cell, and the other electrode is used to measure the resulting change in membrane potential in a connected neighboring cell. Because the two cells are electrically coupled, current injection leads to a change in the membrane potential of both cells (**Figure 1A**). Alternatively, dual-electrode whole-cell voltage clamp can also be used to measure gap junctional coupling; in this configuration, inducing a change in membrane potential between the two cells drives an electrical current through the gap junctions (Spray et al., 1979). Electrophysiological recording has millisecond resolution, picoampere current detection sensitivity, and the ability to measure conductance and rectification, both of which are important properties of electrical synapses in neurons. However, this method has obvious limitations, including the need for relatively high technical expertise, specialized equipment, high invasiveness due to disruption of the cell membrane integrity, relatively low throughput, and one-off recording. Moreover, this method by itself cannot discriminate distinct cell types, which is particularly problematic given the heterogeneous nature of the nervous system. In addition, the recordings are usually performed at the cell body, which does not take into account the subcellular localization of gap junctions, particularly in neurons and other cell types with complex morphology.

## Transfer of Tracers

To assay the gap junction communications, tracers including fluorescent dyes, and bioactive small molecules can be introduced to one cell or a group of cells. The diffusion of tracers from the primary targeted cells to other connected cells reflects the gap junctional couplings.

### Microinjection of the Tracer

The injection of a tracer, followed by measuring its transfer, is usually the first step in identifying the location and morphology of cells within a tissue. Because small molecules can pass freely through gap junctions, the diffusion of an injected small tracer molecule can be used to measure gap junctional coupling between cells (**Figure 1B**). With respect to the study of gap junction–mediated communication, the most commonly used fluorescent dye is Lucifer Yellow, with a molecular weight of 457 Da (Stewart, 1978), and the most commonly used bioactive small molecule is biocytin, with a molecular weight of 372 (Horikawa and Armstrong, 1988). The transfer (i.e., diffusion) of an injected tracer to neighboring cells can be observed either directly (in the case of a fluorescent dye) or *post hoc* using immunohistochemistry (in the case of a bioactive small molecule). The tracers used in these experiments are not membrane-permeable, thereby reducing non-specific diffusion through the cell membrane. This method is technically easier to perform compared to electrophysiology, which requires multiple electrodes and a sophisticated recording setup. However, it still lacks cell type specificity, requires the microelectrode, and the dye diffusion is irreversible, thus preventing the ability to study gap junctions repeatedly in the same cells. Moreover, the injection process requires either mechanical pressure or iontophoresis, and immunohistochemistry takes a relatively long time, thus reducing both the temporal resolution and the throughput.

### Scrape Loading of the Tracer

In addition to the one-by-one injection, the tracer can be introduced into a large population of cells via the scrape (McNeil et al., 1984). Cultured cells in one layer are incubated with gap junction-permeable but cell membrane impermeable dyes as mentioned above and are scraped by a needle or a scalpel. Dye molecules therefore get into wounded cells, and can further diffuse to adjacent cells that are intact but coupled with the scraped cells by gap junctions (**Figure 1C**; el-Fouly et al., 1987). The scrape loading/dye transfer method is the easiest one to implement among all methods discussed here. Because of its simplicity, gap junctional communications were evaluated using this method in many cell types, including fibroblasts (Azzam et al., 2001), germ cells in testis (Decrouy et al., 2004), and astrocytes (Giaume and McCarthy, 1996). The limitations of this method include that it is mostly effective in adherent cells and therefore mainly applied in cultured cells or tissue slices *in vitro*. The scraping procedure in conjunction of cell fixation protocol offers qualitative rather than quantitative data for gap junctional connections.

### Fluorescence Redistribution/Recovery After Photobleaching (FRAP)

To overcome the high invasiveness and technical expertise associated with microelectrode-based methods, Wade et al. (1986) developed the gap-FRAP technique (**Figure 1D**), an all-optical strategy used to study gap junctions. Rather than injecting fluorescent molecules into individual cells, cultured cells are incubated with membrane-permeable fluorescein-AM; upon entering the cell, the ester bond is hydrolyzed by intracellular esterases, leaving the hydrophilic fluorescein molecule trapped within the cell (Rotman and Papermaster, 1966). After an intense focused laser is used to bleach the fluorescein in one cell, the bleached fluorescein molecules and the unbleached fluorescein molecules in neighboring cells diffuse through the gap junctions, leading to the recovery of fluorescence in the original bleached cell. Compared to the methods described above, FRAP is less invasive and easier to perform. Importantly, this method provides both qualitative and quantitative information regarding the strength of the gap junctions, which is reflected by the kinetics of fluorescence recovery (Lee et al., 1995; Soroceanu et al., 2001). This method also provides satisfactory temporal resolution, as the photobleaching can be performed extremely rapidly using a high-power laser (Lippincott-Schwartz et al., 2003). One potential drawback of FRAP is that the intense laser illumination may damage the cell. In addition, in order to quantitatively characterize the FRAP kinetics which reflect the strength of the gap junctional communication, the recovery event needs to be monitored until the fluorescence recover to the plateau, which takes much longer time than the half-time for recovery (Lippincott-Schwartz et al., 2003). This requirement makes FRAP not suitable for measuring very fast dynamics of the gap junctions as can be done by electrophysiological recording (Abbaci et al., 2008). Finally, similar to tracer tracking methods, FRAP by itself lacks cell type specificity, constraining its application mainly to homogenous cell cultures.

### Local Activation of a Molecular Fluorescent Probe (LAMP)

To avoid potential phototoxicity associated with photobleaching while still leaving the cell intact, Dakin et al. (2005) developed LAMP, which uses the caged fluorescent dye NPE-HCCC2-AM (**Figure 1E**; Zhao et al., 2004). After the cell is loaded as described above for NPE-HCCC2-AM, UV illumination is used to uncage NPE-HCCC2 in specific cells and release HCCC2, which has a molecular weight of 450 Da and emits blue fluorescence. The uncaged HCCC2 can then diffuse to neighboring cells connected via gap junctions. In a sense, LAMP is a combination between FRAP and tracer tracking in that it generates a fluorescent signal (the “tracer”) in one cell and then tracks the movement of the tracer through gap junctions, while maintaining cell integrity. In this respect, LAMP has the combined advantages of both methods in that it is non-invasive, provides quantitative data, and has relatively high temporal resolution. In addition, LAMP allows for multicolor imaging, as the uncaging of NPE-HCCC2 requires a small pulse of UV light and is therefore compatible with fluorescent indicators in the visible spectrum (Dakin et al., 2005; Dakin and Li, 2006; Abbaci et al., 2008). This method can be improved further by incorporating caged fluorescent molecules with higher uncaging efficiency and a more penetrable red-shifted emission spectrum. Unfortunately, LAMP still requires loading of an exogenous dye, which limits its applications in *in vivo* preparations. Moreover, uncaging of NPE-HCCC2 is irreversible, making it less suitable for studying the dynamics of gap junctions repeatedly over a prolonged period of time.

## Hybrid Approaches Combined with Genetic Tools

In order to obtain more cell type-specific information, genetically encoded proteins can be incorporated into the method being used to map gap junctions; this is particularly important for studying gap junctions in a specific cell population within the heterogeneous central nervous system.

### Esterase-Ester Pair

This enzyme-substrate pair has been used successfully to map gap junctions (**Figure 1F**). The enzyme is expressed in specific cell types; the substrate, which is water-soluble, membrane-permeable, unaffected by endogenous enzymes but catalyzed by the ectopically expressed enzyme, is able to diffuse through gap junctions. One example of this approach is the highly selective esterase-ester pair developed by Tian et al. (2012). In this approach, they synthesized a series of esters that fluoresce upon hydrolysis, and identified one substrate (called “substrate **6**”) that was stable in several different cell types in a range of species, including flies, rodents, and humans. They also identified porcine liver esterase (PLE) (Lange et al., 2001) as the most potent at catalyzing the hydrolysis of substrate **6** and used the PLE-substrate **6** pair to map the distribution of gap junctions. PLE hydrolyzes substrate **6** to produce a fluorescent product, and the diffusion of this fluorescent product causes fluorescence in the cells that were connected via gap junctions to the PLE-expressing cells (**Figure 1F**). Thanks to genetic manipulation, this strategy provides higher cell type specificity using a relatively simple approach. The bio-specificity of substrate **6** ensures that this strategy can be used in a wide variety of organisms and cell types; however, it is still possible that endogenous esterases can cause a non-specific background signal under certain conditions. Thus, the system can be optimized by modifying the PLE enzyme and/or the substrate, or by identifying a more bioorthogonal enzyme-substrate pair (Sletten and Bertozzi, 2009; Ritter et al., 2015). At the same time, robust control experiments (for example, using knockout models) are an essential step in testing for non-specific background due to endogenous enzymes (Qiao and Sanes, 2015). Another drawback of this method is the relatively low temporal resolution, which requires up to 30 min of incubation in the substrate, thereby limiting its value in terms of studying the dynamics and regulation of gap junctions (Tian et al., 2012). In addition, this method has only been tested in cultured cell lines, and its feasibility in primary cells (e.g., neurons and cardiomyocytes) and *in vivo* applications has not been investigated.

### Transporter-Substrate Pairs

An alternative strategy is to use a transporter-substrate pair in which a genetically encoded transporter is expressed in one cell, which then transports a fluorescent substrate into the cytoplasm; diffusion of the fluorescent substrate to neighboring cells indicates the presence and distribution of gap junctions. In 2015, Qiao and Sanes reported the use of human Pept2 (a peptide transporter) (Biegel et al., 2006) and the AMCA-labeled dipeptide β-Ala-Lys (βALA, the substrate) (Dieck et al., 1999) as a strategy for mapping gap junctions (**Figure 1G**; Qiao and Sanes, 2015). Using this innovative tool, they successfully mapped functional gap junctions in cultured HEK293 cells and quantified the diffusion properties of βALA, which reflects the strength of the gap junctions. Taking advantage of the CreER system and sparse labeling in Pept2 knockout mice, they then confirmed the presence of gap junctional communication between J-RGCs (a subset of retinal ganglion cells) and amacrine cells in the mouse retina (Bloomfield and Volgyi, 2009; Hoshi and Mills, 2009; Volgyi et al., 2009), and they demonstrated the presence of gap junctions in horizontal cells. Importantly, the authors also characterized the light-dependent electrical coupling of horizontal cells by mapping the pattern of gap junctional communication before and after illumination with light (Xin and Bloomfield, 1999). Thus, the Pept2-βALA pair provides a powerful tool for mapping the distribution and strength of gap junction connectivity both in cultured cells and in an *ex vivo* retinal preparation. On the other hand, a clear drawback associated with this method is that the temporal resolution (which is on the order of hours) is not sufficient to track the dynamics of the strength of gap junction connections.

### Genetically Encoded Fluorescent Sensors/Optogenetics Combined With Patch-Clamp Recording

Genetically encoded fluorescent sensors provide another means to map gap junctions by monitoring the concentration change of a chemical during diffusion through gap junctions. In 2016, Kang and Baker reported the development of a novel genetically encoded fluorescent sensor called Pado, which can be used to track the diffusion of protons through gap junctions (**Figure 1H**; Kang and Baker, 2016). Pado is a dual-function protein created by fusing an engineered voltage-gated proton channel from *Clonorchis sinesis* with a pH-sensitive fluorescent protein (Super Ecliptic pHluorin 227A, or SE227A) (Jin et al., 2012). To demonstrate proof-of-principle, Kang and Baker expressed Pado in HEK293 cells, then used the whole-cell patch-clamp technique to depolarize one cell. The change in voltage opened the voltage-gated proton channels, facilitating the efflux of protons from the cell and creating an electrochemical gradient between this cell and neighboring cells connected via gap junctions. Protons then diffused from the neighboring cells down this electrochemical gradient, and the change in SE227A fluorescence was detected in both the clamped cell and the adjacent cells. While this method is promising, the data should be taken carefully and some calibrations allowing for quantitative analysis should be performed. A similar strategy utilizing a hybrid calcium indicator Calcium Green FlAsH could also enable detection of gap junctional couplings, by monitoring the intercellular propagation of calcium waves in gap junction coupled cells (Tour et al., 2007). Given that Calcium Green FlAsH needs to be applied exogenously, further improvements can be achieved by using pure genetically encoded calcium indicators such as GCaMP6 (Chen et al., 2013).

Given the electrical properties of gap junctions, optogenetics is yet another useful tool for mapping gap junctions, as an electrical signal generated by light-activated channelrhodopsins (Nagel et al., 2003, 2005) can propagate to coupled cells and be detected using patch clamp. Recently, Wang et al. (2014) combined an improved version of the channelrhodopsin ChIEF (Lin et al., 2009) with electrophysiology in order to map gap junction connections in the *Drosophila* olfactory system (**Figure 1I**). They performed patch-clamp recordings on cholinergic projection neurons (mPNs) while expressing ChIEF in mediolateral antennocerebral tract projection neurons (mIPNs) labeled by *Mz699-Gal4* (Ito et al., 1997). Applying blue laser illumination to the mIPNs induced depolarization of some mPNs; this effect was not altered by the nicotinic receptor antagonist mecamylamine but was sensitive to the *shakB^2^* mutation (which affects innexin-8) (Thomas and Wyman, 1984; Phelan et al., 1996; Zhang et al., 1999; Song and Tanouye, 2006), leading to the conclusion that mPNs and mIPNs are electrically coupled. The finding that blocking cholinergic receptors had no effect on the mIPN-mPN coupling indicates that when using this tool, it is important to distinguish chemical and electrical synapses using genetics and/or pharmacology, as ChIEF induced depolarization of presynaptic neurons can drive postsynaptic responses in both chemical and electrical synapses. Moreover, unlike the dual-electrode whole-cell patch-clamp technique, the ChIEF-based method is unidirectional and cannot be used to identify rectifying gap junctions. To overcome this limitation, a light-gated chloride pump such as the Halorhodopsin isolated from *Natronomonas* (NpHR) (Han and Boyden, 2007; Zhang et al., 2007) can be used to hyperpolarize the presynaptic terminal, thereby reversing the direction of the current across the electrical synapse.

Compared to previous methods, these two strategies (exemplified by Pado and ChIEF) do not require an exogenously applied substrate, which simplifies the experimental protocol and makes them more feasible for use in *in vivo* applications. In addition, because they have relatively faster kinetics (on the order of milliseconds to seconds), these methods can be used to collect repeated measurements, which is essential for studying the dynamics of the strength of gap junctional connections at high temporal resolution. On the other hand, these approaches require the use of glass micropipettes, reducing their throughput. Moreover, one needs to block chemical synapses when using ChIEF to detect electrical synapses, which may alter the normal state of the nervous system.

## Perspectives

Gap junctions play an extremely important role in mediating cell-cell communication, and their distribution and dynamics are essential for maintaining normal physiological function and homeostasis. Although researchers have been able to link genetic mutations with these conditions, identifying precisely which cell populations are affected by these mutations has been far more difficult. In a more physiological context, single-cell transcriptomics has revealed that both neurons and glia are more heterogeneous than previously believed (Lake et al., 2016; Tasic et al., 2016). In addition, connexins and innexins are encoded by multiple genes, giving rise to a wide diversity of gap junctions. For example, the mouse and human genomes contain 20 and 21 connexin-encoding genes, respectively (Sohl and Willecke, 2003), and the *Caenorhabditis elegans* and *Drosophila melanogaster* genomes contain 25 and 8 innexin-coding genes, respectively (Starich et al., 2001; Stebbings et al., 2002). Therefore, investigating the function of gap junctions in distinct cell types and in an isoform-specific manner remains extremely challenging. To overcome these challenges, new methods providing improved genetic specificity, high spatial resolution, and functionally relevant temporal resolution are urgently needed. Ideally, these methods should be non-invasive and technically simple to perform, thereby facilitating their use in *in vivo* applications, allowing researchers to study gap junctions in a more physiological setting.

In principle, using genetically encoded tools provides a possible solution. For example, the PLE-ester and Pept2-βALA strategies discussed above eliminate the need to manually manipulate the cells with glass pipettes, while providing the advantages associated with fluorescence imaging (Tian et al., 2012; Qiao and Sanes, 2015). On the other hand, the patch-clamp–based Pado and ChIEF strategies provide faster kinetics and do not require an exogenous substrate, making the background signal easier to control by regulating the expression level (Wang et al., 2014; Kang and Baker, 2016). However, each of these methods includes a non-genetically encoded component (e.g., an exogenous substrate or whole-cell patch-clamp recording), which have inherent limitations as discussed above.

**FIGURE 2:**
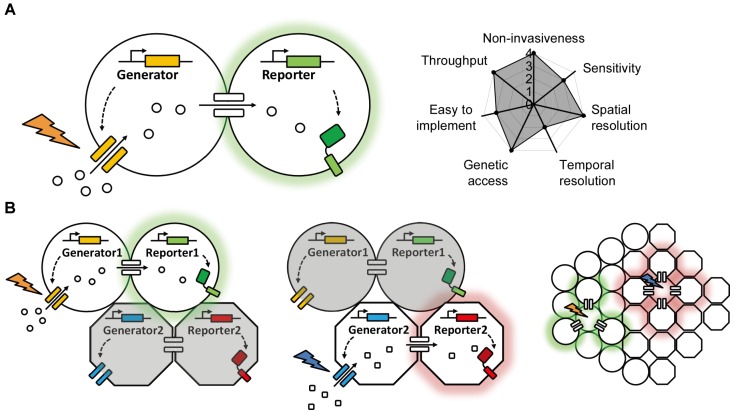
A proposed ideal optogenetics-based system for mapping gap junctions. **(A)** The principle behind the proposed optogenetics-based system shown on the left with its theoretical performance index on the right, similar to **Figure 1**. **(B)** A proposed multiplex, optogenetic system for mapping gap junction using two pairs of bio-orthogonal generators and reporters and its application in an intact tissue with heterogeneous cell types.

The vast majority of genetically encoded methods used to date are based on the diffusion of target molecules such as esters, ions, peptides, or synthetic dyes through gap junctions. In each case, the electrochemical gradient that drives this diffusion is generated exogenously (e.g., by patch clamp, tracer introduction, or substrate application), and the chemical transfer is usually detected using fluorescent probes. Thus, we can summarize the entire system as consisting of a “generator” and a “reporter”; the generator produces the electrochemical gradient between coupled cells, and the reporter reports the transfer of molecules through the gap junctions (**Figure 2A**). In a system comprised exclusively of genetically encoded optogenetics-based components, both the generator and the reporter would be proteins (e.g., a light-activated channel or transporter and a fluorescent sensor). In this idealized system, the generator would be controlled by light and would use the cel’s endogenous ions or chemicals to generate the electrochemical gradient, and the reporter would sense the change in concentration and change its fluorescence intensity. This non-invasive optogenetics-based system could be used to control and image a large number of cells simultaneously, and the background fluorescence could be minimized greatly by controlling the expression of the generator and reporter. More importantly, multicolor imaging could be achieved—at least in theory—by using a combination of generators with non-overlapping action wavelengths and/or reporters with non-overlapping excitation and emission spectra (**Figure 2B**). Given the wide range of clear benefits associated with this approach, genetically encoded optogenetics represents one of the most promising strategies for studying gap junctions in the future.

## Author Contributions

YL, AD, and SL conceived and wrote the manuscript.

## Conflict of Interest Statement

The authors declare that the research was conducted in the absence of any commercial or financial relationships that could be construed as a potential conflict of interest.
